# Development of human cartilage circadian rhythm in a stem cell-chondrogenesis model

**DOI:** 10.7150/thno.70893

**Published:** 2022-05-13

**Authors:** Mark A Naven, Leo A.H. Zeef, Shiyang Li, Paul A Humphreys, Christopher A Smith, Dharshika Pathiranage, Stuart Cain, Steven Woods, Nicola Bates, Manting Au, Chunyi Wen, Susan J Kimber, Qing-Jun Meng

**Affiliations:** 1 Wellcome Centre for Cell Matrix Research, Faculty of Biology, Medicine and Health, University of Manchester, Oxford Road, Manchester, UK; 2 Division of Cell Matrix Biology and Regenerative Medicine, School of Biological Sciences, Faculty of Biology, Medicine and Health, University of Manchester, Manchester Academic Health Science Centre, Manchester, UK; 3 Bioinformatics Core Facility, Faculty of Biology, Medicine and Health, University of Manchester, Oxford Road, Manchester, UK; 4 Department of Biomedical Engineering, The Hong Kong Polytechnic University, Hung Hom, Hong Kong, China

**Keywords:** Human stem cells, chondrogenesis, circadian rhythm, development, osteoarthritis

## Abstract

The circadian clock in murine articular cartilage is a critical temporal regulatory mechanism for tissue homeostasis and osteoarthritis. However, translation of these findings into humans has been hampered by the difficulty in obtaining circadian time series human cartilage tissues. As such, a suitable model is needed to understand the initiation and regulation of circadian rhythms in human cartilage.

**Methods:** We used a chondrogenic differentiation protocol on human embryonic stem cells (hESCs) as a proxy for early human chondrocyte development. Chondrogenesis was validated using histology and expression of pluripotency and differentiation markers. The molecular circadian clock was tracked in real time by lentiviral transduction of human clock gene luciferase reporters. Differentiation-coupled gene expression was assessed by RNAseq and differential expression analysis.

**Results:** hESCs lacked functional circadian rhythms in clock gene expression. During chondrogenic differentiation, there was an expected reduction of pluripotency markers (e.g., *NANOG* and *OCT4*) and a significant increase of chondrogenic genes (*SOX9*, *COL2A1* and *ACAN*). Histology of the 3D cartilage pellets at day 21 showed a matrix architecture resembling human cartilage, with readily detectable core clock proteins (BMAL1, CLOCK and PER2). Importantly, the circadian clocks in differentiating hESCs were activated between day 11 (end of the 2D stage) and day 21 (10 days after 3D differentiation) in the chondrogenic differentiation protocol. RNA sequencing revealed striking differentiation coupled changes in the expression levels of most clock genes and a range of clock regulators.

**Conclusions:** The circadian clock is gradually activated through a differentiation-coupled mechanism in a human chondrogenesis model. These findings provide a human 3D chondrogenic model to investigate the role of the circadian clock during normal homeostasis and in diseases such as osteoarthritis.

## Introduction

Degeneration of the articular cartilage is a hallmark of osteoarthritis (OA), a prevalent joint disease, causing severe pain and disability, with currently no cure 1-4. Better understanding of the normal physiology of chondrocytes and how they become dysregulated during ageing or injury could inform the development of treatments for OA. One emerging area of importance to cartilage biology is the discovery of circadian rhythms in articular cartilage and chondrocytes. Several groups have reported circadian rhythm disruptions as one of the common pathways in aged or osteoarthritic cartilage in rodents 5-10. Similar rhythm disruption has been observed in human clinical samples as well as retrospective cohort studies 5-10. Cell autonomous circadian rhythms in mouse articular cartilage drive the temporal expression of approximately 10% of genes expressed in cartilage. Many of these, such as key chondrogenic molecules *ACAN*, *COL2A1* and *SOX9*, are major players in cartilage homeostasis 5, 10. During ageing and inflammation, the robust circadian oscillations of genes become dampened. Circadian clock genes have been shown to play critical roles in cartilage homeostasis and OA through use of mouse models with deletions or mutations in *Bmal1*, *Clock* and *Cry2* 5, 6, 9, 10.

In contrast to the well-defined rodent models which allow real-time tracking of clock gene/protein dynamics, studies of circadian clocks in human cartilage have been more limited. Investigations are largely restricted to the expression of individual clock components in archived clinical samples or the dysregulation of clock pathways in comparative RNAseq datasets 11-14. This is partly because obtaining normal human cartilage tissue is particularly challenging, let alone obtaining a circadian time series of samples. One alternative model is human embryonic stem cells (hESCs) which can be differentiated towards a chondrogenic lineage 15-17. This model not only provides a potential source of cells capable of meeting clinical requirements for a future regenerative therapy, but also serves as a valuable model for mechanistic studies of human cartilage development and OA disease progression. We have recently refined a chondrogenic differentiation protocol (RAPID) from hESCs, showing increased expression of *SOX5, SOX6*, *SOX9*, *COL2A1* and *ACAN* during the first 11 days of differentiation 15-17. 3D pellets from these chondrogenic cells form large, relatively uniform structures with strong Alcian blue staining and peripheral Lubricin. The distinct proteoglycan-rich pericellular matrix organised into lacunae-like structures and different zones, which more closely resemble mature articular cartilage than in some earlier protocols 17. Using this model, we set out to address when the circadian clock first initiates during chondrogenic differentiation in human chondrogenesis. *In vivo*, a functional circadian rhythm is crucial to matrix expression and cell functionality 5, 6, 9, 10. As such, understanding this process in chondrogenesis is vital to develop novel protocols to generate better quality cartilage. This will have wider implications in the field, particularly in regard to tissue engineering, and providing more accurate *in vitro* models for diseases.

## Methods

### hESCs culture

hESCs line MAN13 18 was cultured in feeder-free culture conditions in 6 well plates coated in vitronectin (5 µg/mL) (Thermo) in mTeSR1 medium (Stem Cell Technologies). Cells were passaged at 80% confluence by detachment with 0.5 mM EDTA in PBS. The culture medium was changed every two days.

### RAPID differentiation protocol and chondrogenic pellet culture

hESCs were differentiated as previously described 17. Briefly, 100,000 hESCs/ cm^2^ were split into fibronectin coated 16.6 µg/mL (Millipore) 6-well plates and cultured until 70% confluency to start the protocol. To start, mTeSR1 medium was removed and directed differentiation basal medium (DDBM) was added for chondrogenic differentiation. Chondrogenic pellets were generated as described previously 17. Briefly, day 11 chondroprogenitors were detached from adherent culture by TryplE express (Thermo) and centrifuged at 300x RCF in day 11 culture medium for 5 minutes to compact cells. The cells were cultured for a further three days at 37 °C in 5% CO_2_. Day 11 medium was changed at day 11 + 3 for chondrogenic-basal medium (CM) containing GDF5 (40 ng/ml), TGFβ3 (10 ng/ml) (both Peprotech) and BMP2 (0.5 X EC 66) (R&D systems). Pellets were cultured for up to 28 days with medium changed every 3 days.

### Western Blotting

Cells were lysed in RIPA buffer (Sigma) containing protease inhibitor cocktail (Promega). Protein concentration was quantified using Pierce BCA (Thermo). A total of 30 µg of protein per sample was heated at 95 °C for 10 minutes in fluorescence compatible sample buffer (Thermo). Samples were loaded on 10% SDS PAGE gels (Invitrogen). Separated proteins were transferred to nitrocellulose membranes using iBlot2™ (Thermo) and then submerged in Intercept blocking buffer - PBS (LI-COR) before primary antibody incubation. Antibody binding was detected utilising IRDye fluorescent secondary antibodies (LI-COR). Images were taken using Odyssey CLx scanner. Protein quantification was calculated relative to alpha tubulin internal control using FIJI analysis software.

### qRT-PCR transcriptional analysis

Cell samples were taken in both the 2D and 3D part of the protocol, as described previously 15,17. Samples were lysed using RLT buffer and RNA extracted using RNeasy Qiagen kit. Isolated RNA was converted to cDNA using high-capacity cDNA reverse transcription kit (Thermo). Quantitative real-time polymerase chain reaction (qRT-PCR) assay was conducted using PowerUp™ SYBR™ Green (Life Technologies). qRT-PCR data were analysed using the 2^-ΔCt^ method and normalised to GAPDH reference gene.

### Immunohistochemistry and histology

Chondrogenic pellets were fixed in 4% PFA overnight at 4 °C before automated tissue processing (Leica ASP300s) and embedding in PFFE wax blocks. Samples were cut to a thickness of 5 µm. Histological analysis (H&E, Alcian blue and Picrosirius red staining) of 3D chondrogenic pellet sections were performed in the Histology Facility at the University of Manchester, according to the standard protocols. For IHC, sections were subject to antigen retrieval by heating in citrate buffer pH 6.0 at 95°C for 10 minutes, then permeabilised using 1% (v/v) Triton X-100 (Sigma) in PBS for 30 minutes and blocked in 10% goat serum in PBS for 30 minutes. Rabbit primary antibodies for BMAL1 (Merck Millipore #AB2204), PER2 (Merck Millipore #AB2202) and CLOCK (Merck Millipore #AB2203) were generated and validated by Le Sauter et al 19 and added to pellet sections at 4 °C overnight. After washing, slides were incubated with biotinylated goat anti-rabbit secondary antibodies (Vector) for 2 hours at 4 °C. Nuclei were stained using 4′,6-diamidino-2-phenylindole (DAPI, life technologies #D1306). Sections were then incubated with horseradish peroxidase solution (Vectorlabs) for 1 hour at room temperature, then visualised with DAB (3,3′-Diaminobenzidine) tablets (Merck) for 3 minutes. The samples were then washed, dehydrated and mounted with coverslips.

Human cartilage tissues were obtained by orthopaedic surgeons in Queen Mary Hospital of Hong Kong under ethics committee approval (REC ref13/EM/0388) and processed in histology laboratory in the Hong Kong Polytechnic University. In brief, the cartilage specimens were punched from the lateral tibial plateau where they were relatively well preserved in osteoarthritic knee joints compared to the medial side. The specimens were then decalcified and embedded in wax for routine histological examinations. Five µm thick sections were cut and stained with hematoxylin and eosin (H&E) or Safranin O and examined under bright field light microscopy.

### Immunocytochemistry

Cells were seeded and allowed to attach for 24 hours. Cells were fixed using 4% paraformaldehyde (Sigma) for 15 minutes, permeabilised for 5 minutes using 0.1% Triton-X (Sigma) solution, blocked in 10% goat serum for 30 minutes and incubated with primary antibodies (diluted 1:500 in PBS containing 1% goat serum for 12 hours at 4 ºC on a benchtop rocker. Antibodies against CLOCK #AB2203 and BMAL1 #AB2204 were kindly provided by David Weaver and are available from Merck Millipore. Antibodies for PER1 (Rabbit #13463-1-AP) and CRY1 (rabbit #13474-1-AP) were from ProteinTech, antibodies for NANOG (rabbit IgG #4903) and OCT4 (Mouse IgG #75463 and rabbit IgG #2890) were from Cell Signalling. Secondary antibodies AlexaFluor plus 488 (Goat anti-rabbit IgG # A32731) or AlexaFluor plus 594 (Goat anti-mouse IgG # A32742) (Life Technologies) were added for 2 hours at room temperature. Nuclei were stained using 4′,6-diamidino-2-phenylindole (DAPI, Life Technologies #D1306). Fluorescent images were taken using a Zeiss Axioimager D2 microscope equipped with a Coolsnap HQ2 camera (Photometrics) and micromanager software version 1.4.23.

### Lentiviral packaging of reporter constructs

The lentiviral vector containing a Luciferase reporter that we described previously 10 was modified to include a T2A linker and an improved variant of the green fluorescent protein (TurboGFP) sequence to allow FACS sorting. Promoter sequences of human *BMAL1* and *PER2* genes were synthesised (Thermo Fisher) and subcloned into this vector upstream of the sequence encoding the luciferase enzyme (vector maps in Figure S1) using the NEBuilder® HiFi DNA Assembly (NEB). HEK293T cells were transfected with virus packaging vectors psPAX2 and pMD2.G (VSV) and reporter plasmid, either *hPER2*-Luc or *hBMAL1*-Luc, using calcium chloride transfection. Twenty-four hours post transfection viral titre was increased by addition of 10 mM sodium butyrate (Millipore) for 4 hours. Medium was collected after 48 hours and filtered through a 0.2 μm filter before viral particle concentration at 6000 g (J-25 Avanti centrifuge and F10BA-6x500y rotor) at 4 °C for 12 hours. The resulting pellet was resuspended in 10ml PBS and was again concentrated at 50,000 g for 90 minutes using a SW40-Ti rotor (Beckman Coulter). The pellet was resuspended in 200 µl of ice-cold PBS and stored at -80 °C until use.

### Bioluminescence analysis

hESCs were either transduced with lentivirus at the stem cell stage or were differentiated for 8 days before lentiviral transduction. Cells were treated with 100 nM dexamethasone (Sigma) for 1 hour and then the medium was changed to recording medium. Recording medium for 2D cells (serum and phenol-free DMEM) (Thermo) contained 0.1 mM luciferin, 10 mM HEPES, 305 mg/L sodium bicarbonate, 50mM B-mercaptoethanol, 200 mM L-Glutamine, B27 and Insulin-transferrin-selenium (Thermo). Recording medium for 3D pellets were optimised and contained 200 mM L-Glutamine, 5 mg/mL Ascorbic-2-phosphate, Proline 4 mg/mL and ITS. Cells at 2D stages were imaged using an Alligator bioluminescence machine Cairn Research using iXon Ultra EMCCD camera. Pellets at 3D stages were immobilised in a thin layer of low melting point 1% agarose to prevent movement and imaged by the Olympus Luminoview LV200 microscope with a cooled Hamamatsu Image EM C9100-13 EM-CCD camera (Olympus).

### RNA sequencing and analysis

RNA isolated from samples was analysed for quality utilising a TapeStation 4200 (Agilent) according to the manufacturer's instructions. RNA concentration was determined utilising Qubit analysis (Qubit 4™ Thermo). RNA was labelled with TruSeq RNA library preparation kit (Illumina) and analysed using HiSeq4000 system (Illumina). Unmapped paired-end sequences from an Illumina HiSeq4000 sequencer were tested by FastQC (http://www.bioinformatics.babraham.ac.uk/projects/fastqc/). Sequence adapters were removed, and reads were quality trimmed using Trimmomatic_0.39. The reads were mapped against the reference human genome (hg38) and counts per gene were calculated using annotation from GENCODE 36 (http://www.gencodegenes.org/) using STAR_2.7.7a. The gene list was annotated using BioMart software. Normalisation, Principal Components Analysis, and differential expression across the 3 time points were calculated with the Likelihood ratio test of DESeq2_1.18.1. Genes with an adjusted p < 0.1 and log2FC > 1 or < -1 were deemed significant. Heatmaps were generated with ComplexHeatmap in R (https://www.R-project.org/). Raw data were deposited in EMBL-EBI Array Express (accession number E-MTAB-11758).

### Statistical analysis

Statistical analysis was run using GraphPad Prism 9 (version 9.0.2). Results were assessed for normal distribution using Shapiro-Wilk test, parametric data were analysed using one way ANOVA with Tukey's correction and non-parametric data were analysed using Friedman test and Dunn's correction. Differences between groups were considered significant when p < 0.05. For all graphs shown, error bars represent ± standard error of the mean. *p < 0.05, **p < 0.01, ***p < 0.001 and ****p < 0.0001.

## Results

### MAN13 human embryonic stem cells maintain expression of key pluripotency markers

**Figure 1:**
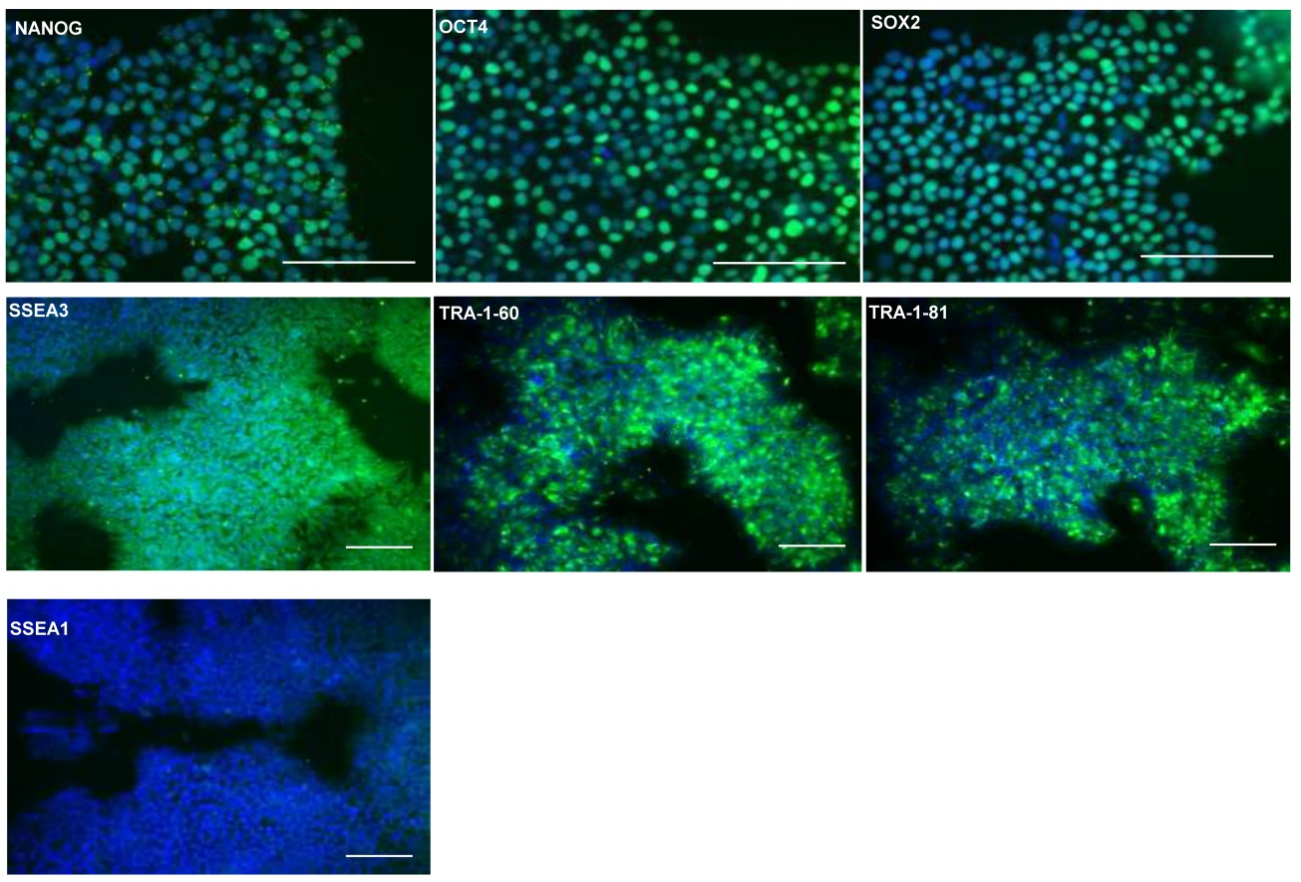
** Confirmation of expression of stem cell markers in hESC (MAN13) cells.** Representative immunofluorescence of pluripotency markers in MAN13 hESCs with primary antibodies against: NANOG, OCT4, SOX2, SSEA3, SSEA1, TRA1-81 and TRA1-60. Goat anti-rabbit Alexa Fluor plus 488 antibody was used to detect primary antibodies. DAPI was used for nuclear staining. (N = 3; scale bars = 100 µm).

Previously, seven clinical grade hESCs lines were generated according to Good Manufacturing Practice (GMP) standards, including MAN13 cells which were chosen for directed chondrogenic differentiation in this study 18. Expression of pluripotency associated markers were confirmed by immunoflourescence (IF) staining for transcription factors NANOG, octamer-binding protein 4 (OCT4), SRY-box transcription factor 2 (SOX2) and surface markers Stage Specific Embryonic Antigen 3 (SSEA3), Trafalgar (TRA)-1-81 and TRA-1-60. Nuclear expression of transcription factors NANOG, OCT4 and SOX2 was confirmed in the hESCs (Figure 1). Moreover, these cells expressed surface markers associated with pluripotency, such as SSEA3, TRA-1-81 and TRA-1-60, but not SSEA1, an early differentiation marker for pluripotent stem cells (Figure 1).

### Successful differentiation of MAN13 cells towards a chondrogenic lineage

**Figure 2:**
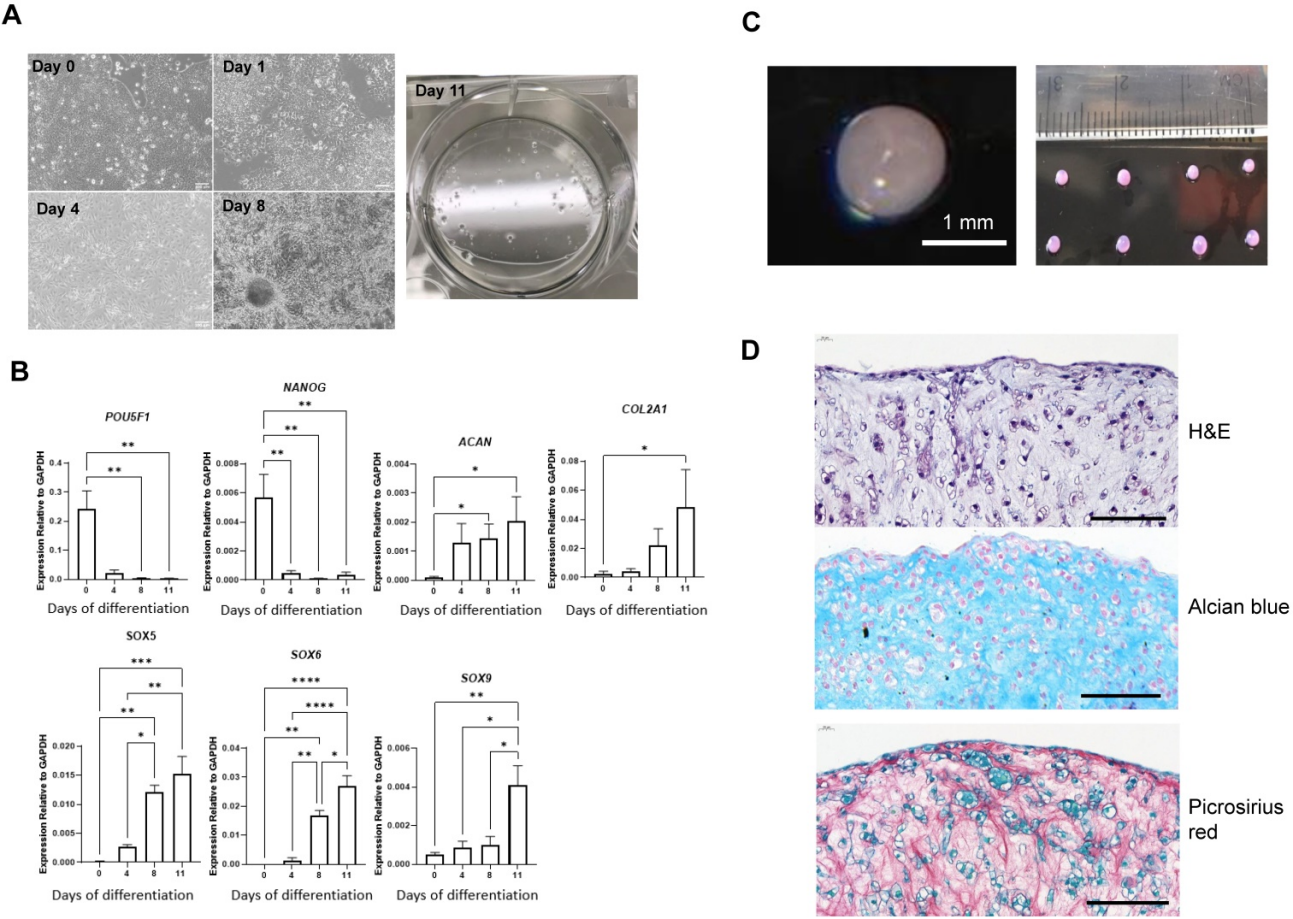
** Differentiation of MAN13 stem cells into pre-chondrocytes through the RAPID protocol. (A).** Cell morphology showing MAN13 hESCs at day 1, 4, 8 and 11 with the appearance of cell aggregates. **(B).** qPCR analysis of pluripotency-associated and chondrogenic marker genes during the 2D stage (11 days) of the RAPID differentiation protocol. N = 3-5. *, P < 0.05; **, P < 0.01; ***, P < 0.001; ****, P < 0.0001. **(C).** Photographs of 3D chondrogenic pellets. **(D).** Histology evaluation of 3D pellets (Haematoxylin and Eosin, Alcian blue and Picrosirius red). N = 3, scale bars = 100 µm.

A revised chondrogenic differentiation protocol which replaced growth factors with small molecules and closely followed the endogenous route of cartilage development through the lateral plate mesoderm (designated RAPID) was developed recently 17. This showed better chondrogenic gene expression and consistent formation of 3D chondrogenic pellets. MAN13 hESCs were directed to a chondrogenic lineage according to the RAPID protocol, involving 2D differentiation over 11 days in adherent culture followed by a 3D differentiation as cell aggregates in suspension (pellets) 17. The morphology of the cells underwent significant changes throughout 2D differentiation. MAN13 hESCs displayed classical pluripotent cell morphology with large nucleus-to-cytoplasm ratio and grew in tightly condensed colonies (Figure 2A). After differentiation, cells gradually lost their typical condensed colonies. At day 4, a monolayer of highly uniform cells was seen. At day 11 (end of 2D stage), cells spontaneously formed aggregates (Figure 2A) as seen previously 16.

Gene expression analysis showed that the mRNA levels of key pluripotency genes* POU5F1 (a.k.a. OCT4)* and *NANOG* were significantly decreased by day 11. Expression of two of the most abundant cartilage matrix genes *COL2A1* and *ACAN* was significantly increased at day 11 of the protocol (Figure 2B). *SOX5, SOX6* and *SOX9,* essential genes in mesenchymal condensation and chondrocyte development 20, all showed gradual increase in their expression during the differentiation process (Figure 2B). SOX9 is a master regulator of chondrogenesis and acts in concert with SOX5 and SOX6 to drive chondrogenic differentiation 20.

Pellets were formed by centrifugation from 1 million cells at day 11 of the RAPID protocol. Over a period of 24 hours, these cells self-assembled into roughly spherical clusters. Continued culture yielded 3D, highly uniform spheres with smooth reflective surfaces and a similar translucent appearance to articular cartilage (Figure 2C). Chondrocytes and extracellular matrix are organised in a characteristic manner in articular cartilage. Histological analysis using H&E staining revealed that by day 21 (10 days in 3D) of the protocol, cells resided within an extracellular matrix. Cartilage lacunae were observed comparable to those in adult human cartilage (Figure 2D, Figure S2). Alcian blue staining confirmed that the pellet matrix contained glycosaminoglycans, as in human cartilage tissues (Figure 2D). Picrosirius red staining revealed collagen fibre networks throughout the pellet structure (Figure 2D). Thus, the RAPID protocol produces a cell population with chondrogenic gene expression and an extracellular matrix structure closely resembling native human cartilage.

### MAN13 human embryonic stem cells lack functional circadian rhythms

**Figure 3:**
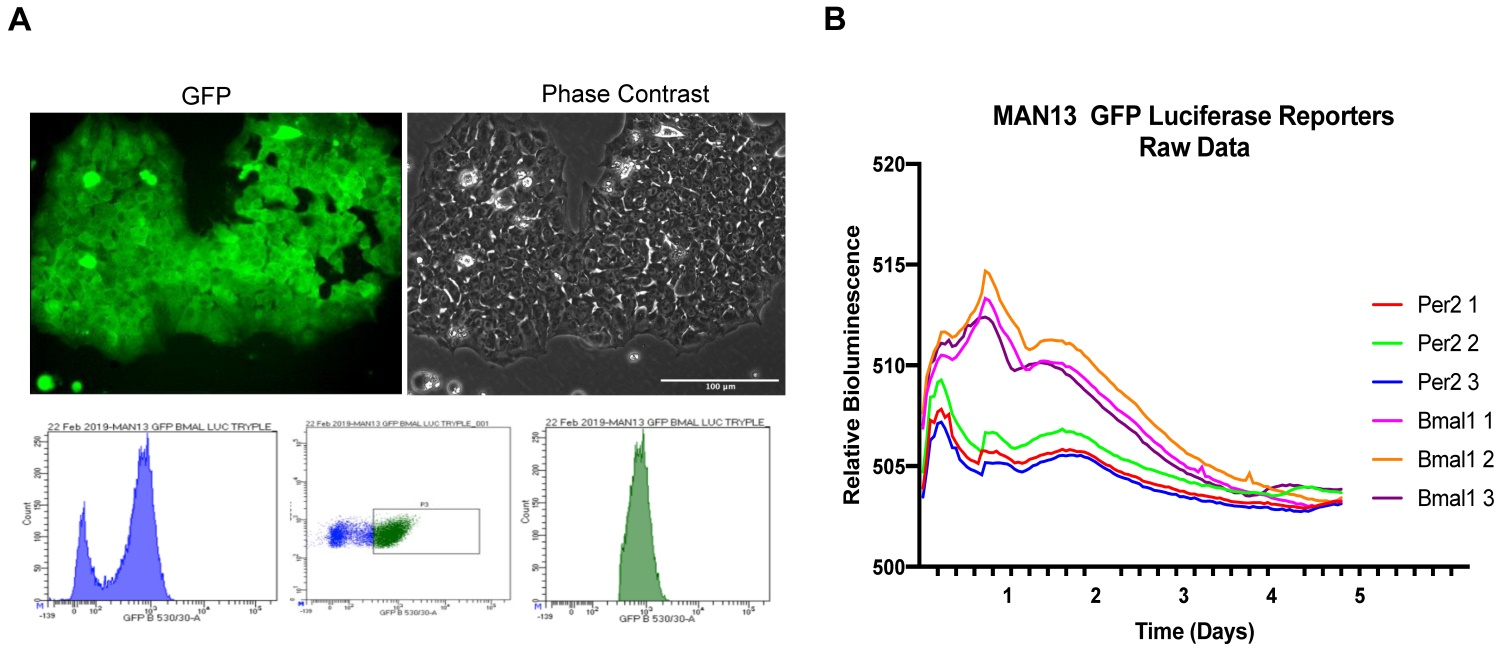
** Lack of circadian rhythm in hESC (MAN13) cells. (A).** Fluorescence and phase contrast images after lentiviral transduction of MAN13 cells with *hBMAL1*-Luc or *hPER2*-Luc plasmids, scale bar = 100 µm. Cells were sorted by FACS based on GFP signal intensity (bottom panel). **(B).** Bioluminescence traces of *hBMAL1*-Luc and *hPER2*-Luc reporters in dexamethasone treated GFP sorted MAN13 cells (N = 3 for each reporter).

To determine if these cells maintained a functional circadian rhythm, the MAN13 hESCs were transduced with lentiviral reporters expressing luciferase under the control of human *PER2* and *BMAL1* gene promoters. These constructs also constitutively express GFP, allowing monitoring of transduction efficiency and FACS sorting of positive cells. As a positive control, single colonies of TC28a2 human chondrocyte-derived cells transduced with the same reporters showed robust 24-hour oscillations (Figure S3). Successful transduction of MAN13 cells was confirmed by GFP expression and cells were FACS sorted to isolate reporter-expressing population (Figure 3A). These MAN13 cells were treated with Dexamethasone and subjected to bioluminescence imaging using the Alligator system. MAN13 cells exhibited bioluminescence but did not display any evidence of circadian oscillation from either of the two clock gene reporters (Figure 3B). These findings indicate the absence of a functional circadian clock machinery to drive rhythmic transcription in hESCs.

### Initiation of the circadian rhythm during chondrogenic differentiation

**Figure 4:**
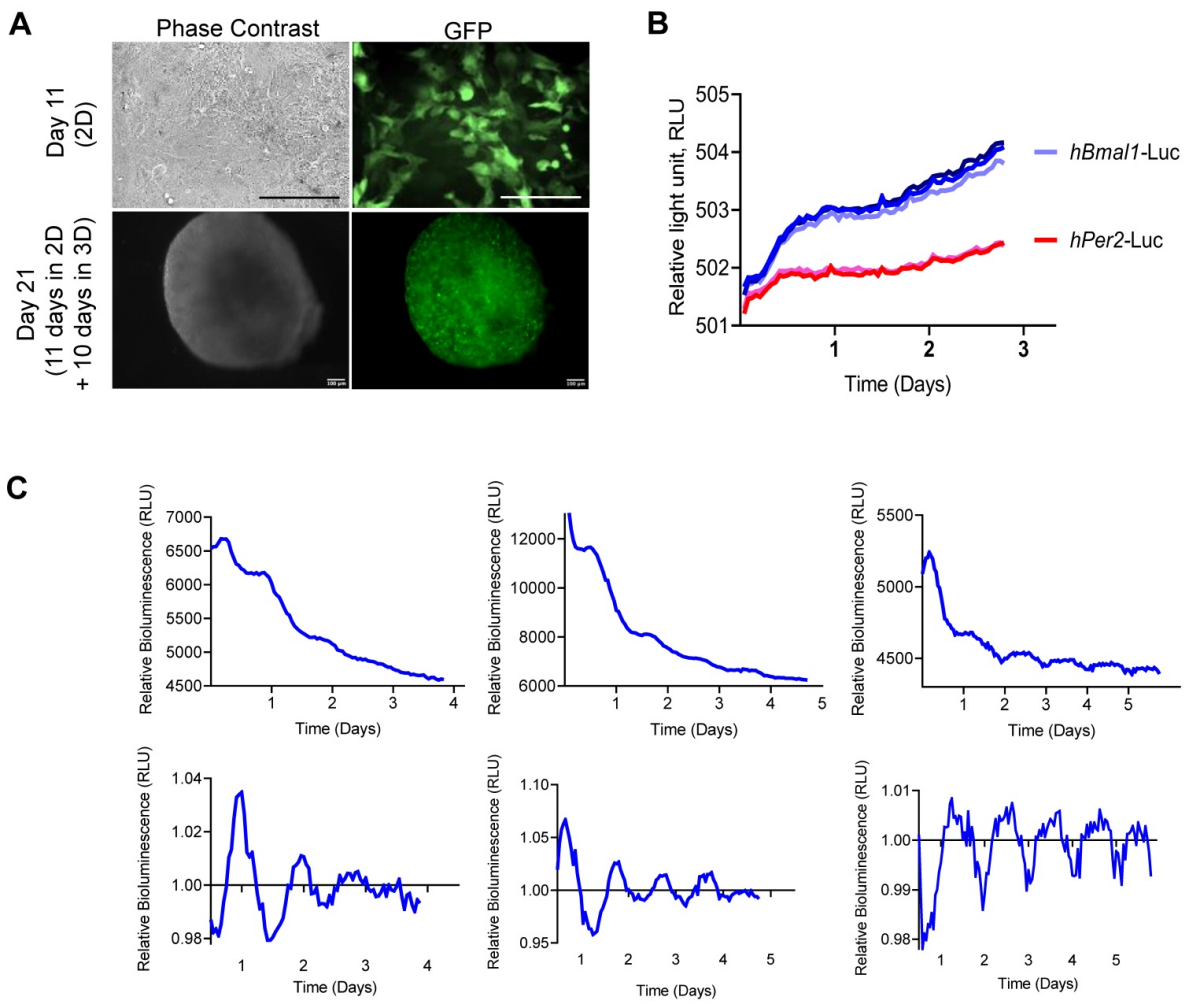
** Gradual initiation of circadian rhythm during hESC (MAN13) chondrogenic differentiation. (A).** Successful transduction of clock reporters (*hBMAL1*-Luc or *hPER2*-Luc) into cells at day 11 of the differentiation protocol (2D monolayer) or day 21 (10 days in 3D). N = 3, scale bars = 100 µm.** (B).** At Day 11 (2D), cells were treated with Dex and bioluminescence signals were recorded by the Alligator system. Raw data (Relative light unit, RLU) from a whole well of cells were plotted as intensity over time. Representative traces of 3 independent differentiation runs, with 2-3 technical replicates within each run (N = 3). **(C).** At Day 21 (3D), pellets transduced with *hBMAL1*-Luc were synchronised by Dex and bioluminescence signals were recorded by LV200 imaging system. Three independent differentiation runs (N = 3). Raw data (Relative light unit, RLU) from a region of interest encompassing a whole pellet were plotted as intensity over time. 24 hour moving average of the raw data was plotted in the bottom panels.

It is currently unknown when the circadian clock first activates during skeletogenesis. It is difficult to study this in human foetal development. Therefore, the *in vitro* hESC-chondrogenesis model provides an opportunity to investigate human cartilage development and the ontogenesis of circadian rhythms. To determine if the circadian clock is activated during chondrogenic differentiation of hESCs, MAN13 cells were transduced with lentiviral *hBMAL1*-Luc or *hPER2*-Luc reporters at day 8 of the protocol and synchronized at day 11 for clock recording (Figure 4A). Real time bioluminescence analysis revealed lack of circadian oscillations for both reporters at day 11 of the 2D protocol (Figure 4B).

**Figure 5:**
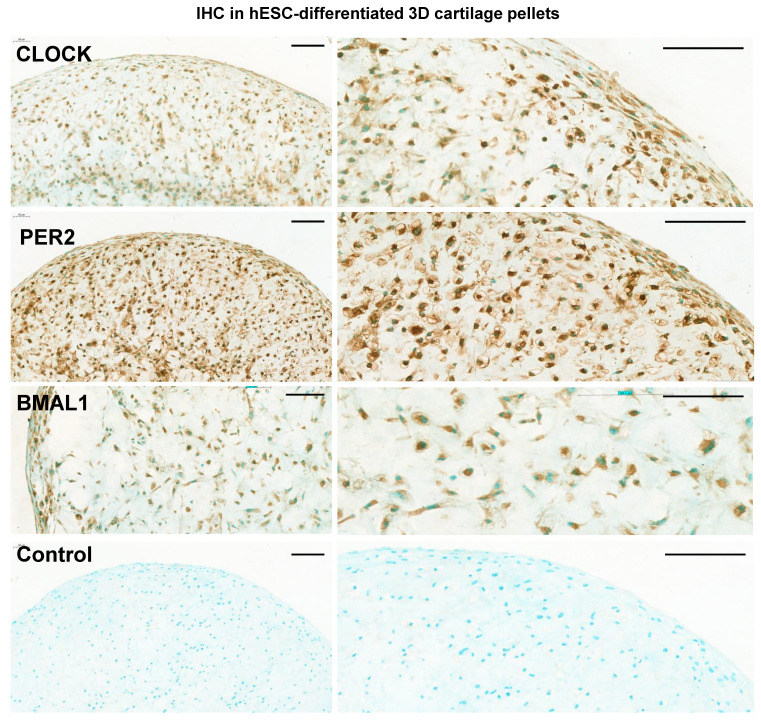
** Expression of clock proteins in 3D chondrogenic pellets.** Representative immunohistochemistry of chondrogenic pellets at day 11 (2D) + day 10 (3D) stained with primary antibodies to clock factors CLOCK, PER2 and BMAL1. N = 3. Negative control sections were incubated without the primary antibody. Panels on the right show higher magnifications of images on the left panels. Scale bars = 100 µm.

Next, *hBMAL1*-Luc reporter cells were centrifuged and cultured in 3D; differentiation continued for a further 10 days to form 3D pellets (Figure 4A). For bioluminescence analysis the pellets were imaged for circadian rhythmicity using the LV200 microscope (Olympus). Strong bioluminescence signals and a clearly detectable pattern of approximately 24-hour oscillations of *hBMAL1*-Luc were detected (Figure 4C, n = 3 separate RAPID protocols). Biodare analysis using the Fast Fourier Transform Non-Linear Least Squares algorithm (FFT NLS) of normalized data showed approximately 24-hour oscillations (p < 0.00001). Consistent with the presence of circadian rhythms, CLOCK, PER2 and BMAL1 proteins were expressed in cells of the chondrogenic pellets at day 21 (Figure 5 and Figure S4). These results support a differentiation coupled activation mechanism of the chondrocyte circadian clock in a human stem cell chondrogenesis model.

**Figure 6:**
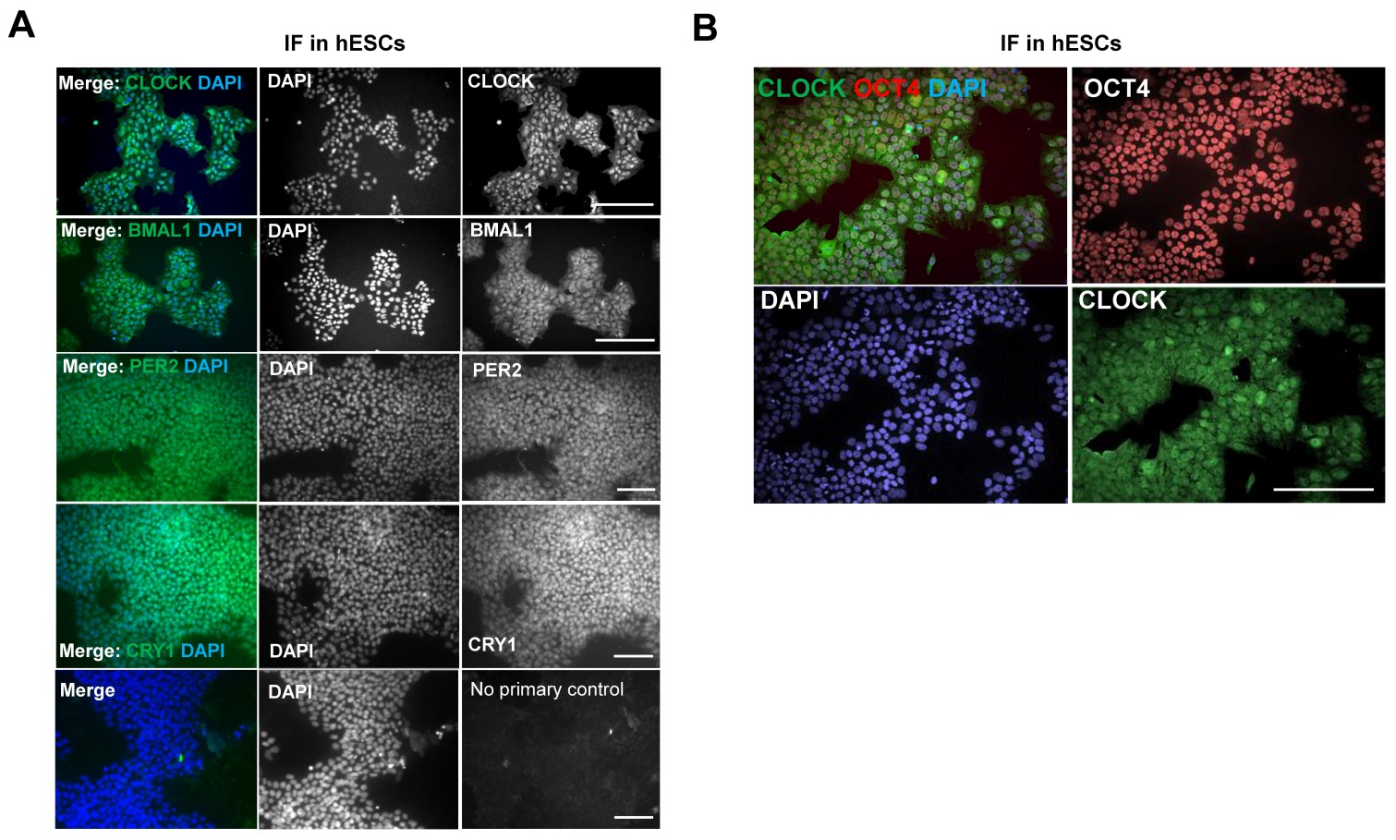
** Expression of clock proteins are readily detectable in hESCs. (A).** Representative IF of clock factors in hESCs. MAN13 cells were fixed and incubated with antibodies against CLOCK, BMAL1, PER2 and CRY1, followed by Alexa Fluor plus 488 antibody. Nuclei were stained with DAPI. Scale bars represent 100 μm. Representative images from N = 3 except CRY1 (N = 2). **(B).** Co-expression of CLOCK (in green) and OCT4 (in red) proteins in MAN13 hESCs by IF. Nuclei were stained with DAPI. Scale bar = 100 μm. Representative images of N = 2.

Surprisingly, using IF, protein levels of CLOCK, BMAL1, PER2 and CRY1 could all be detected in MAN13 cells, both within the nucleus and cytoplasm (Figure 6A). This is in contrast to mouse ESCs where CLOCK protein was not detected 21. Further analysis of CLOCK by western blotting confirmed its expression in human MAN13 cells and revealed its co-expression with OCT4 (a marker of pluripotency) in nuclei (Figure S5; Figure 6B). These results suggest that an absence of a functional circadian clock in hESCs is unlikely a result of missing key protein components of the core molecular clock. Instead, a change of stoichiometry of various clock factors, post-translational modifications, epigenetic mechanisms or co-transcription factors may be required for the activation of the circadian clock.

### RNA sequencing reveals transcriptome level changes between the inactive and active stage of clock oscillations in the RAPID protocol

To gain further insights into the mechanisms of clock activation during chondrogenesis, RNA sequencing was conducted to quantify gene expression levels at a transcriptome scale before differentiation, at the end of the 2D stage of differentiation (at day 11) and after a further 10 days of 3D differentiation (day 21) when the clock becomes active. Principal Component Analysis revealed differentiation stage as the biggest factor separating the sample groups (Figure S6A). Differential gene expression analysis revealed that amongst the top 50 genes that showed the most significant variations between the three groups, there was a loss of pluripotency markers *POU5F1 (OCT4)* and *SOX2* during differentiation and an increase of genes associated with cartilage matrix (*CLIP, COMP, DCN* and *FMOD)* (Figure S6B).

**Figure 7:**
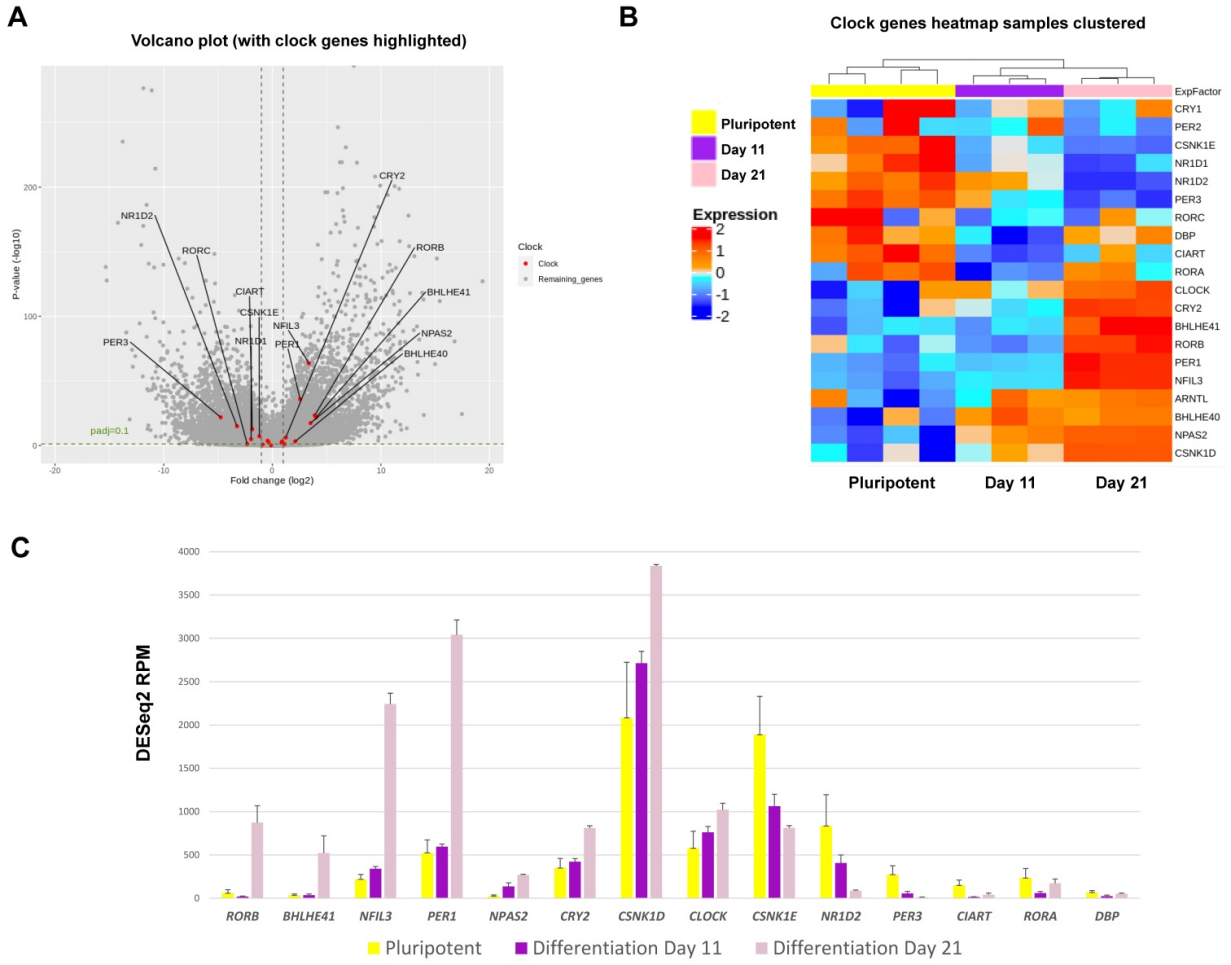
** Expression changes for circadian clock genes as revealed by RNAseq during RAPID chondrogenic differentiation of MAN13 cells. (A).** Volcano plot of differential expression analysis results for all genes whose expression levels followed the chondrogenic developmental stages. Data were plotted against -Log10 p-value. Horizontal dotted lines represent significance level of padj < 0.1. Vertical dotted lines indicate Log2 fold change of -1 and 1 between Day 21 vs. Day 11. Significant clock genes with padj < 0.1 and log2FC > 1 or < -1 were highlighted in red with gene names. **(B).** Heatmap of the clock genes that showed significant variations among the three time points, as revealed by differential expression analysis. **(C).** RNAseq data (DESeq2 normalised counts) for clock genes that showed most significant variations among the three time points (padj < 0.01 and padj < 0.05 between Day 21 vs. Day 11). Mean ± SE, N = 3-4. Between Day 21 and Day 11: padj < 0.05 for *CSNK1E, CLOCK* and *DBP*; padj < 0.01 for *NPAS2* and *CIART*; padj < 0.001 for all the other genes.

**Figure 8:**
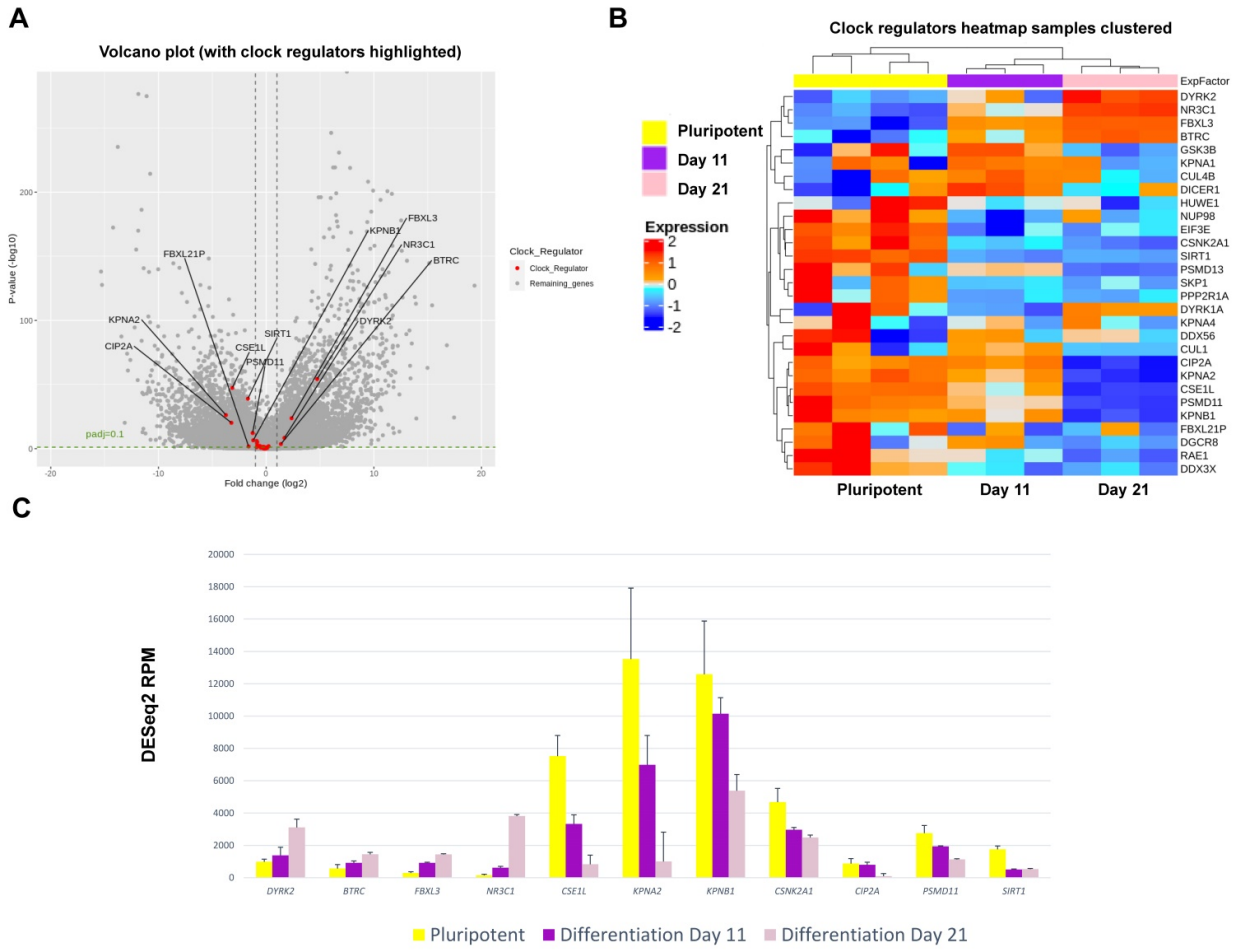
** Expression changes for clock regulator genes as revealed by RNAseq during chondrogenic differentiation of MAN13 cells. (A).** Volcano plot of differential expression analysis showing all differentially expressed genes during the chondrogenic developmental stages. Data were plotted against -Log10 p-value. Horizontal dotted lines represent significance level of padj < 0.1. Vertical dotted lines indicate Log2 fold change of -1 and 1 between Day 21 vs. Day 11. Significant clock regulator genes with padj < 0.1 and log2FC > 1 or log2FC < -1 were highlighted in red with gene names. **(B).** Heatmap of the clock regulator genes that showed significant variations among the three time points, as revealed by differential expression analysis. **(C).** RNAseq data (DESeq2 normalised counts) for clock regulator genes that showed most significant variations in expression levels among the three time points (padj < 0.001). Between Day 21 and Day 11: padj < 0.05 for *CSNK2A1*; not significant for *SIRT1*; padj < 0.001 for all the other genes.

In hESCs, most clock genes were detectable at the transcript level, consistent with IF results. Crucially, several core clock genes including *NPAS2, PER1, CRY2, NFIL3, RORβ, CSNK1δ* and* DEC2* showed significant upregulation during differentiation (Figure 7)*.* In contrast, clock genes *CSNK1ε, NR1D2, PER3, RORα, CIART* and *DBP* were significantly downregulated during chondrogenesis (Figure 7). The dataset also revealed profound differentiation-coupled changes for the expression of proteins reported to regulate the circadian clock (Figure 8). The glucocorticoid receptor *NR3C1* was significantly upregulated between day 11 and day 21, which may suggest that the differentiated cells become more responsive to synchronization via the glucocorticoid receptor 22. F-Box/LRR-repeat protein 3 (*FBLX3*), which regulates the degradation of CRY and PER proteins 23-25, is also increased. Sirtuin1 (*SIRT1*), a histone deacetylase involved in epigenetic regulation of circadian clocks by regulating PER2 deacetylation 26, showed decreased expression with differentiation, especially between pluripotency and differentiation day 11. This decrease in SIRT1 was also seen in previous work 27*. CIP2A (PP2A),* a protein phosphatase reported to regulate PER2 and CLOCK phosphorylation states in *Drosophila* S2 cells 28, 29, showed a significant decrease during differentiation. Karyopherin Subunit alpha 2 (also known as Importin alpha 2) (*KPNA2*) showed decreased expression during differentiation, consistent with earlier reports in mouse ESC differentiation studies 30. Interestingly, nuclear transport proteins Chromosome Segregation 1 like (*CSE1L*), and Karyopherin Subunit Beta 1 (*KPNB1*) were significantly decreased during differentiation. RNA interference (RNAi) targeting these two proteins has been reported to inhibit the circadian clock 31, 32. As such, the profound changes in the expression of clock genes and their regulators support the hypothesis that the clock stoichiometry and/or post-translational modifications may underpin the key events for the initiation of circadian rhythms during cartilage development.

## Discussion

In embryogenesis, articular cartilage is derived from lateral plate (LP) mesoderm through a process called chondrogenesis - the process by which cartilage is formed from condensed mesenchyme tissue, which differentiates into chondroblasts and begins secreting molecules (including aggrecan and collagen type II) that form the extracellular matrix 20, 33, 34. Following the initial chondrogenesis, cartilage growth consists mostly of the transition of immature cartilage to a more mature state 34, 35. During formation of the limb endochondral skeleton a structure called the interzone is formed at the site of joint formation which gives rise to the joint tissues including permanent cartilage 36-38. Investigation of when the circadian clock first activates during skeletogenesis in humans is intrinsically difficult. Recent advances in the stem cell field have made differentiation of hESCs towards pre-chondrocytes a possible model for the study of early cartilage developmental processes and diseases of the skeletal system 39-41. Indeed, the refined RAPID protocol follows the endogenous LP developmental route towards pre-chondrocytes, expressing limb bud markers within 11 days 17. As such, this *in vitro* model is a reasonable reflection of the human chondrocyte development. Using this model, we confirmed the down regulation of pluripotency markers, concomitant with significant increases in chondrogenic genes such as *SOX9*, *SOX6*, *COL2A1* and Aggrecan (*ACAN*) during MAN13-chondrogenic differentiation.

Our data revealed that hESCs lack functional circadian clocks, which is consistent with earlier reports that cells isolated from the inner cell mass from both murine and human embryos do not maintain a circadian rhythm 42-44. Similarly, hiPSCs do not appear to maintain a functional circadian clock 42. The detection of core clock proteins (BMAL1, CLOCK, PER2 and CRY1) in MAN13 hESCs suggests the absence of the circadian clock is not due to missing components of the core molecular clock itself. This is in contrast to mouse ESCs, where it has been shown that the lack of circadian rhythms is mediated by microRNA suppression of CLOCK protein 21. Similar species-specific observations were reported by Dierickx et al who showed expression of CLOCK protein in human pluripotent stem cells but could not detect circadian rhythms 42. These findings suggest additional mechanisms may exist in human stem cells that prevent circadian rhythmicity.

Importantly, our data revealed that the circadian clocks in MAN13 cells were activated between day 11 (end of the 2D stage) and day 21 (10 days after the start of 3D differentiation) of the chondrogenic differentiation protocol, suggesting differentiation-coupled circadian clock activation. Umemura et al analysed 14-28 days of mESCs differentiation and found a differentiation coupled circadian clock activation, mimicking the activation of the clock seen in developing murine hearts 43. This differentiation-coupled activation is comparable to studies of directed differentiation of hESCs towards cardiomyocytes by Dierickx et al, showing gradual activation of the circadian clock after 30 days 42. Alagha et al also reported active circadian clocks in chondrogenic chick limb micromass cultures 45. However, no studies have investigated the ontogenesis of circadian rhythms during chondrogenic differentiation of hESCs in 3D cultures.

Mechanistically, RNA sequencing at day 0 (pluripotent stem cells), day 11 (arrhythmic) and day 21 (rhythmic stage) revealed striking changes in the expression levels of most clock genes. These results support the hypothesis of a change of stoichiometry within the clock machinery. In addition, RNAseq also revealed differentiation-coupled expression of a plethora of molecules that have previously been implicated as key regulators of the molecular circadian clock, including the glucocorticoid receptor (synchronization pathway), the FBLX3 (regulator of CRY stability), SIRT1 (deacetylation of PER2 and BMAL1), PP2A (phosphorylation of PER2 and CLOCK), KPNA2 and CSE1L (nuclear import of clock factors). Therefore, the cell-intrinsic development of the chondrocyte circadian clock seems to be a highly coordinated and multi-faceted process involving a range of clock genes and their regulators.

Early work investigating the ontogenesis of the circadian clock *in vivo* suggested that the circadian rhythm in rodent models activated just prior to birth and continued to develop postnatally 46-49. More recently, robust circadian oscillations of clock gene reporters were observed in developing murine SCN neurons at embryonic day E15.5, and the amplitude continued to increase postnatally 50. In humans, non-invasive analysis of foetal heart rate and movement *in utero* revealed clear diurnal patterns at 22 weeks of pregnancy 51. In a human infant, circadian rhythm of body temperature became apparent 1 week postnatally, a recurrent waking time developed at day 45 (coincided with melatonin increases at sunset) and a stable sleep time did not occur until day 56 52. Collectively, these data suggest that, as is the case in rodent models, the development of human molecular circadian rhythm is most likely a pre-natal event 50. However, the system continues to develop postnatally to drive high amplitude rhythmic outputs in physiology and behavior 53.

Studies in multiple *in vivo* models demonstrate that the foetal circadian clock is likely genetically programmed to develop independently of a maternal circadian clock or a rhythmic environment. In mice carrying compound mutations of clock genes, it has been reported that pups develop normal circadian rhythms despite their arrhythmic mothers 54. In addition, Drosophila born and raised in constant darkness and temperature develop robust circadian rhythms in daily locomotor activity 55. Similarly, zebrafish raised in constant darkness develop circadian clocks without external environmental stimuli, although the cellular clocks are asynchronous 56. Our data provide additional evidence for this notion of cell intrinsic ontogenesis of the cartilage circadian clock.

Taken together, we have investigated the developmental process and regulatory mechanisms of the human cartilage circadian clocks using an established *in vitro* chondrogenic differentiation model from human stem cells. Our data have identified the critical stage when the circadian clocks are activated and revealed novel insights into the gene expression profiles at the stage of circadian rhythm activation during chondrogenesis. Further investigation of circadian timekeeping mechanisms may lead to better understanding of circadian disruption in diseased and ageing cartilage, as well as the development of optimized differentiation protocols for cartilage tissue regeneration.

## Supplementary Material

Supplementary figures.

## Figures and Tables

**Table 1:** Primers

Gene	Forward Primer	Reverse Primer
*GAPDH*	ATGGGGAAGGTGAAGGTCG	TAAAAGCAGCCCTGGTGACC
*ACAN*	TCGAGGACAGCGAGGCC	TCGAGGGTGTAGCGTGTAGAGA
*POU5F1*	AGACCA TCTGCCGCTTTGAG	GCAAGGGCCGCAGCTT
*NANOG*	GGCTCTGTTTTGCTATATCCCCTAA	CATTACGATGCAGCAAATACAAGA
*SOX5*	ATCCCAACTACCATGGCAGCT	TGCAGTTGGAGTGGGCCTA
*SOX6*	GCAGTGATCAACATGTGGCCT	CGCTGTCCCAGTCAGCATCT
*SOX9*	GACTTCCGCGACGTGGAC	GTTGGGCGGCAGGTACTG
*COL2*	GGCAATAGCAGGTTCACGTACA	CGATAACAGTCTTGCCCCACTT
