# Supplementary material for: Development of human cartilage circadian rhythm in a stem cell-chondrogenesis model
Source: Theranostics. 2022 May 13;12(8):3963–76. doi: 10.7150/thno.70893 (PMC9131279; doi:10.7150/thno.70893)
Supplement: Supplementary file 1 — Supplementary figures. [file thnov12p3963s1.pdf]

Development of human cartilage circadian rhythm in a stem cell-chondrogenesis model

Mark A Naven[1,2], Leo A.H. Zeef[3], Shiyang Li[1,2], Paul A Humphreys[2], Christopher A Smith[2], Dharshika Pathiranage[2], Stuart Cain[2], Steven Woods[2], Nicola Bates[2], Manting Au[4], Chunyi Wen[4], *Susan J Kimber[2] and *Qing-Jun Meng[1,2]

Supplementary figure legends:

Fig. S1. Vector maps of new Lentiviral clock gene reporter constructs (*hBMAL1*-Luc and *hPER2*-Luc) containing a GFP sequence after a T2A linker.

Figure S2. Histological staining of human cartilage as controls.

Human cartilage sections were cut to 5 μm and subjected to histological stains. The stains shown are representative examples of N = 3 patients. Scale bar = 50 μm. **(A).** Haematoxylin and Eosin Staining. **(B).** Safranin O staining. The representative images were from a 68 years old female knee osteoarthritis patient.

Fig. S3. Evaluation of clock gene reporters in the TC28a human chondrocyte cell line.

(A). Colony selection schematic. Cells were transduced and then a preliminary selection was run to enrich a polyclonal population of low GFP intensity. A second sort was carried out by seeding single cells into individual wells of 96 well plates. These were expanded for up to 3 months until sufficient cell numbers were obtained. The cells were then split from 96 well plates into T25 flasks and further cultured. The colonies were screened: cells that exhibited minimal changes to morphology and exhibited good circadian reporter rhythm were expanded and cryopreserved. **(B).** Representative fluorescence microscopy image of FACS sorted cells to confirm GFP expression and assess morphology. Cells can be cultured in 3D. Scale bars = 100 μm.

(C). Representative bioluminescence traces of colonies of TC28a cells stably transduced with lentiviral clock reporters.

(D). Normalised data based on C), highlighting the antiphase oscillations of *hBMAL1*-Luc and *hPER2*-Luc reporters in TC28a2 cells (N = 3).

Fig. S4. Validation of antibodies for PER2 and BMAL1 in a human chondrosacoma SW1353 cell line.

The size of PER2 is expected to be > 200 kDa (upper arrow), while the smaller band under 150 kDa is likely the unphosphorylated form of PER2 (lower arrow). The expected size of BMAL1 is 69 kDa.

Fig. S5. Verification of the expression of CLOCK proteins in MAN13 stem cells during chondrogenic differentiation.

Whole cell lysates were collected from MAN13 hESCs on days 0, 4, 8 and 11 of the RAPID protocol. Proteins were separated by molecular weight using bis-tris 10% gels and detected with anti-CLOCK and anti-alpha tubulin primary antibodies followed by GFP- and RFP- labelled secondary antibodies (LI-COR), respectively. Membranes were analysed on LI-COR Odyssey CLx using ImageStudio v4.2 Software.

Fig. S6. Genome-wide changes of gene expression during RAPID differentiation of MAN13 hESCs.

(A). Principal Component Analysis (PCA) of RNAseq data showing separation of the three groups at different developmental stages (time points). **(B).** Heatmap of the top 50 genes that showed most significant variations among the three groups revealed by differential expression analysis. Samples were clustered according to developmental stages indicated by the bar at top of the graph. The graph shows N = 3 (for Day 11 and Day 21) or N = 4 (for pluripotent cells) with replicates in sequential order.

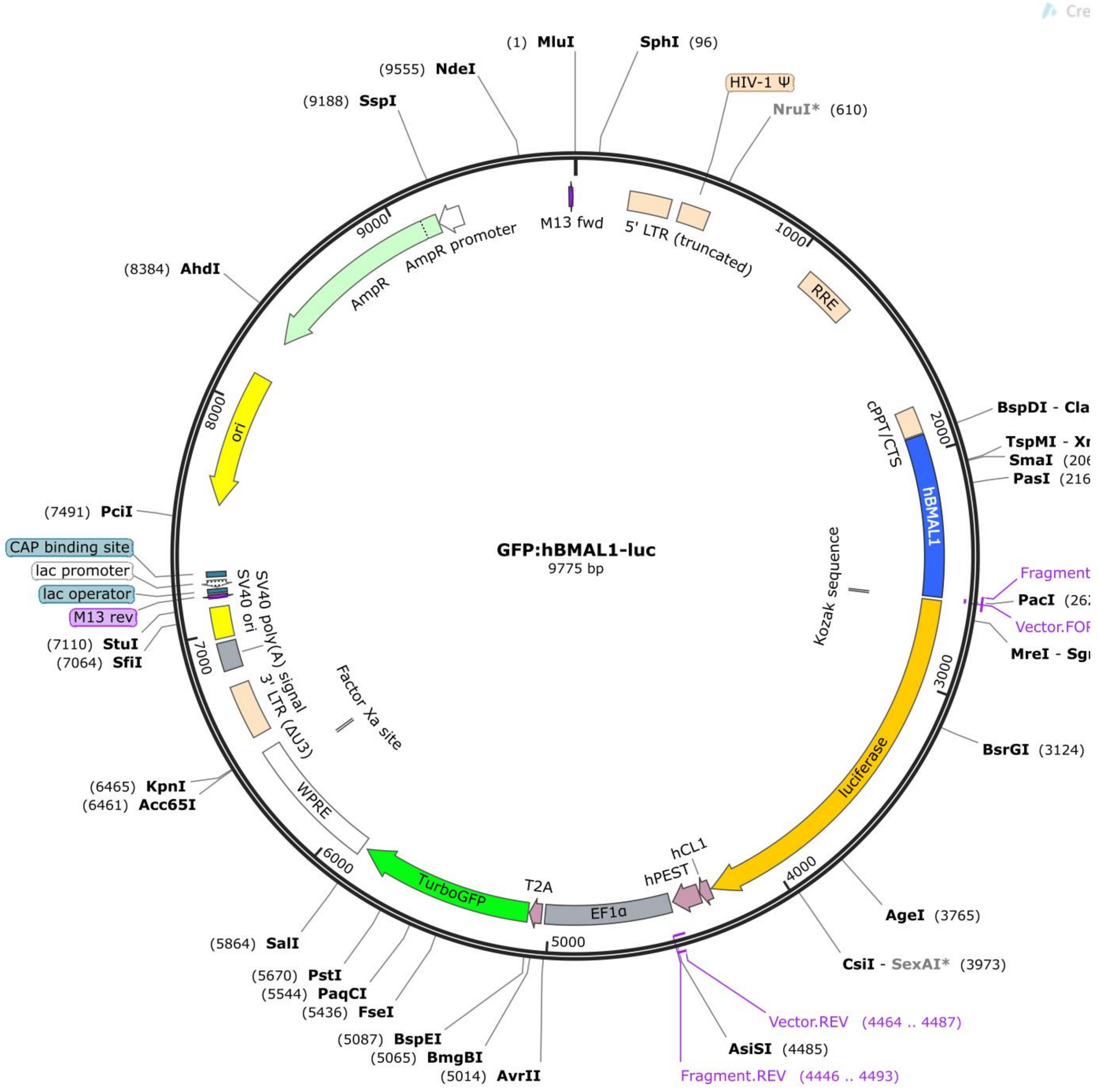

GFP:hBMAL1-luc
9775 bp
(1) MluI
SphI (96)
(9555) NdeI
(9188) SspI
HIV-1 Ψ
NruI* (610)
(8384) AhdI
M13 fwd
5' LTR (truncated)
AmpR promoter
AmpR
RRE
ori
cPPT/CTS
hBMAL1
Kozak sequence
luciferase
BspDI - Cla
TspMI - Xı
SmaI (206
PasI (216
PacI (26:
MreI - Sgı
BsrGI (3124)
AgeI (3765)
CsiI - SexAI* (3973)
Vector.REV (4464 .. 4487)
AsiSI (4485)
Fragment.REV (4446 .. 4493)
hCL1
hPEST
EF1α
T2A
TurboGFP
WPRE
3' LTR (ΔU3)
SV40 poly(A) signal
SV40 ori
Factor Xa site
(7491) PciI
CAP binding site
lac promoter
lac operator
M13 rev
(7110) StuI
(7064) SfiI
(6465) KpnI
(6461) Acc65I
(5864) SalI
(5670) PstI
(5544) PaqCI
(5436) FseI
(5087) BspEI
(5065) BmgBI
(5014) AvrII

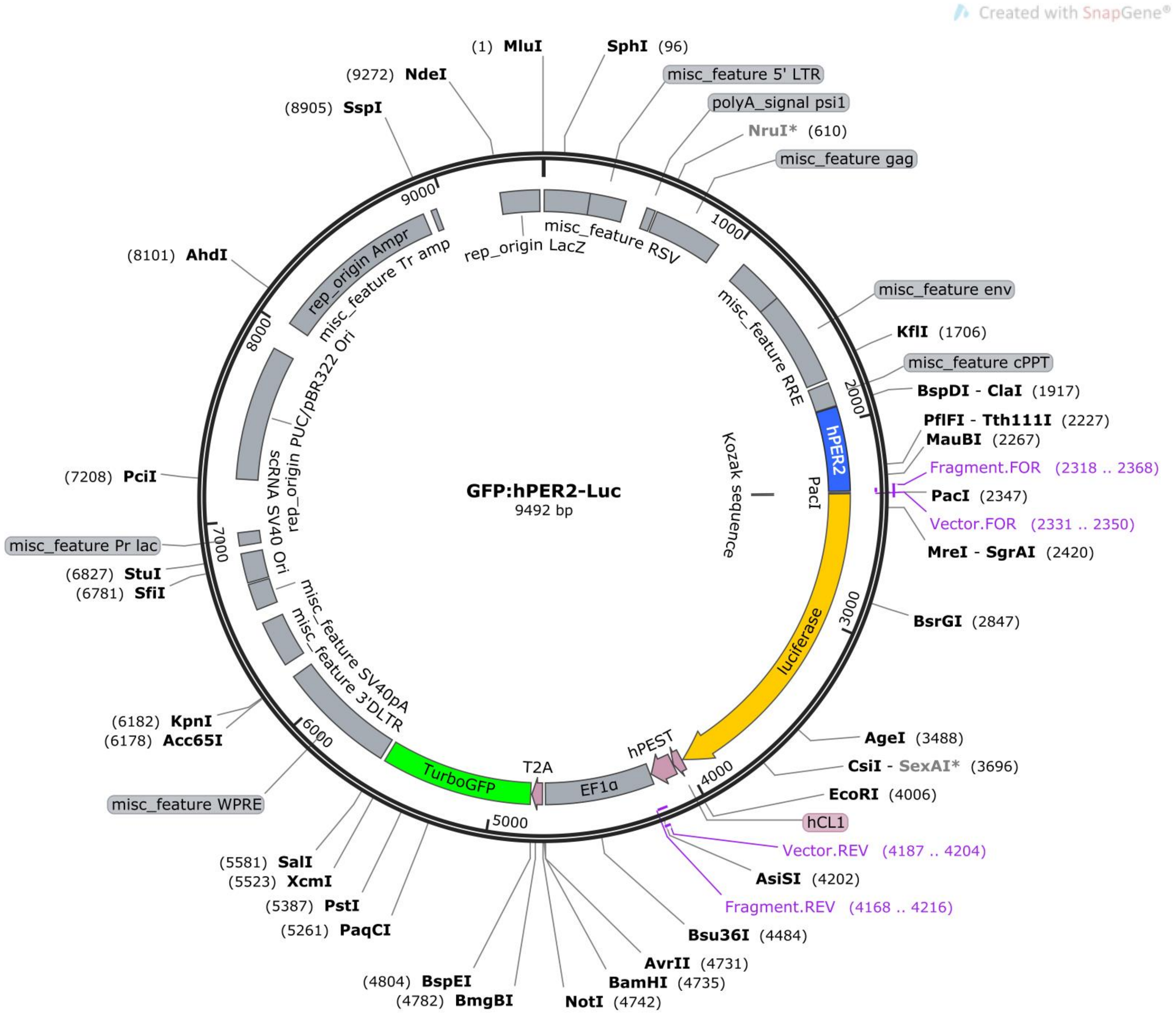

Created with SnapGene®
GFP:hPER2-Luc
9492 bp
(1) MluI
SphI (96)
(9272) NdeI
(8905) SspI
misc_feature 5' LTR
polyA_signal psi1
NruI* (610)
misc_feature gag
(8101) AhdI
rep_origin LacZ
misc_feature RSV
rep_origin Ampr
misc_feature Tr amp
rep_origin PUC/pBR322 Ori
scRNA SV40 Ori
misc_feature RRE
misc_feature env
KflI (1706)
misc_feature cPPT
BspDI - ClaI (1917)
PflFI - Tth111I (2227)
MauBI (2267)
Fragment.FOR (2318 .. 2368)
PacI (2347)
Vector.FOR (2331 .. 2350)
MreI - SgrAI (2420)
hPER2
Kozak sequence
PacI
luciferase
BsrGI (2847)
AgeI (3488)
CsiI - SexAI* (3696)
EcoRI (4006)
hCL1
Vector.REV (4187 .. 4204)
AsiSI (4202)
Fragment.REV (4168 .. 4216)
Bsu36I (4484)
AvrII (4731)
BamHI (4735)
NotI (4742)
(4804) BspEI
(4782) BmgBI
hPEST
EF1α
T2A
TurboGFP
misc_feature SV40pA
misc_feature 3'DLTR
(7208) PciI
misc_feature Pr lac
(6827) StuI
(6781) SfiI
(6182) KpnI
(6178) Acc65I
misc_feature WPRE
(5581) SalI
(5523) XcmI
(5387) PstI
(5261) PaqCI

Figure S1

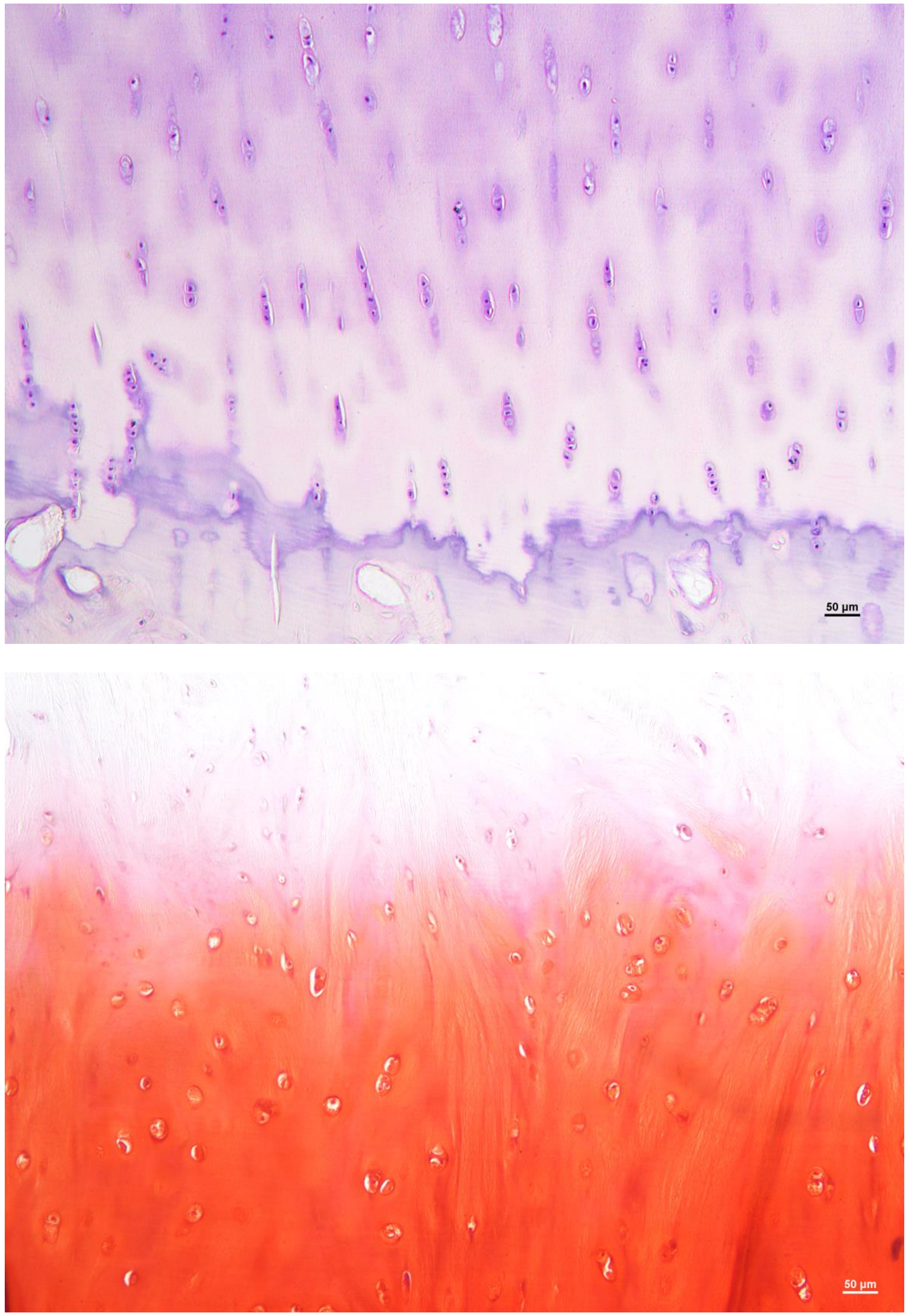

Figure S2

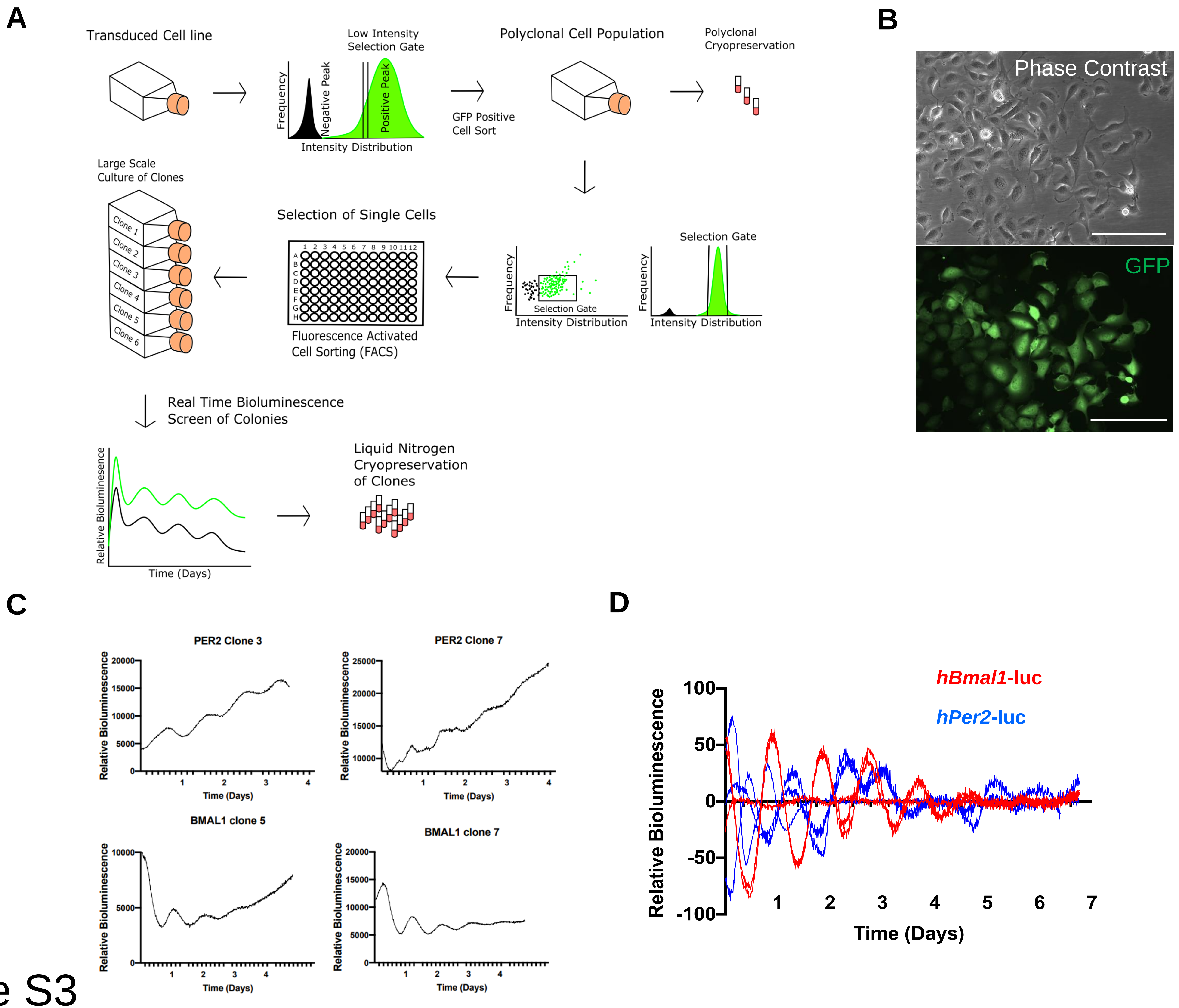
A
Transduced Cell line
Low Intensity Selection Gate
Frequency
Negative Peak
Positive Peak
Intensity Distribution
GFP Positive Cell Sort
Polyclonal Cell Population
Polyclonal Cryopreservation
Large Scale Culture of Clones
Clone 1
Clone 2
Clone 3
Clone 4
Clone 5
Clone 6
Selection of Single Cells
Fluorescence Activated Cell Sorting (FACS)
Selection Gate
Intensity Distribution
Real Time Bioluminescence Screen of Colonies
Relative Bioluminesence
Time (Days)
Liquid Nitrogen Cryopreservation of Clones
B
Phase Contrast
GFP
C
PER2 Clone 3
PER2 Clone 7
BMAL1 clone 5
BMAL1 clone 7
Relative Bioluminescence
Time (Days)
D
hBmal1-luc
hPer2-luc
Relative Bioluminescence
Time (Days)

Figure S3

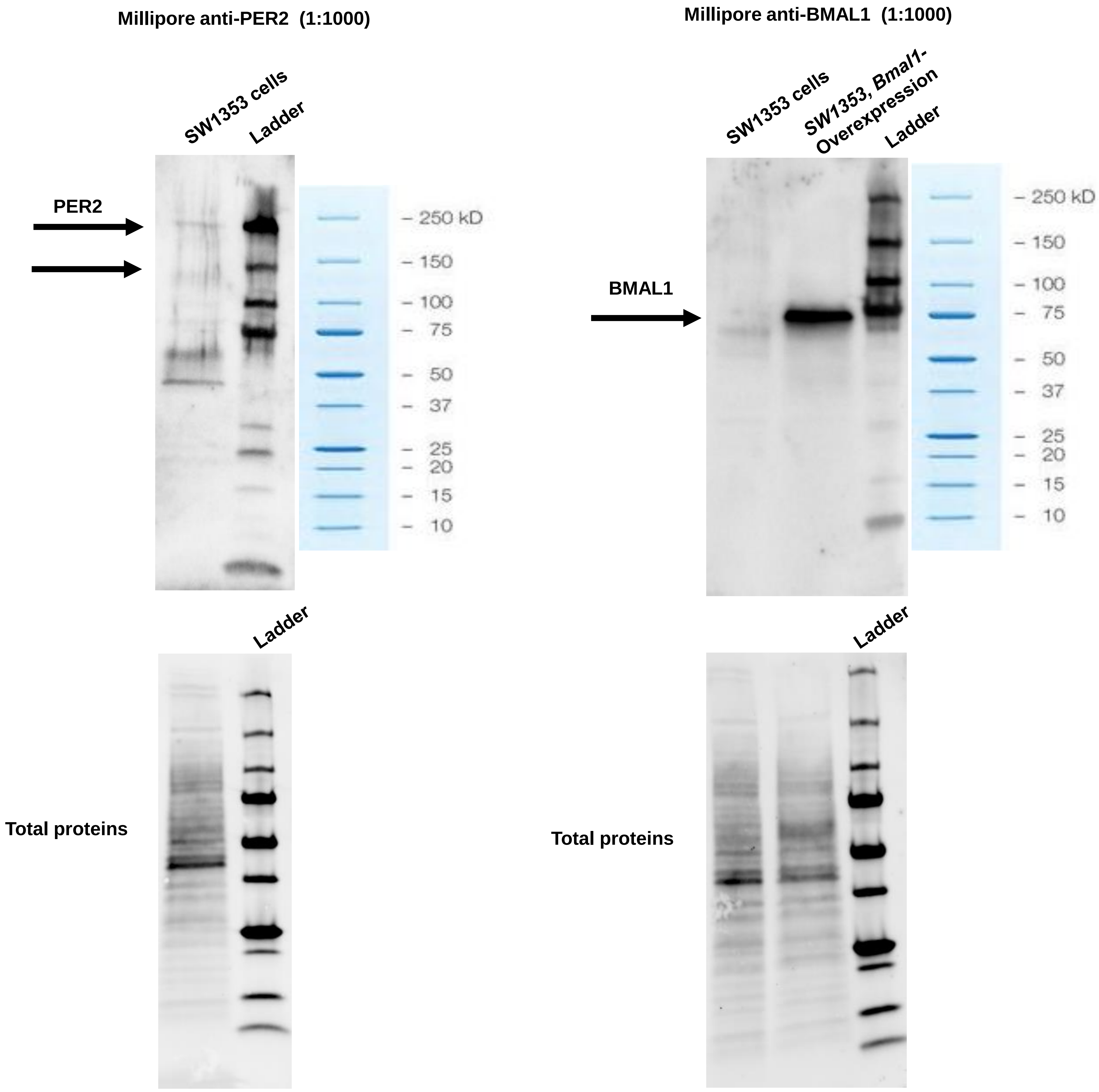
Millipore anti-PER2 (1:1000)
SW1353 cells
Ladder
PER2
– 250 kD
– 150
– 100
– 75
– 50
– 37
– 25
– 20
– 15
– 10
Ladder
Total proteins
Millipore anti-BMAL1 (1:1000)
SW1353 cells
SW1353, Bmal1-Overexpression
Ladder
BMAL1
– 250 kD
– 150
– 100
– 75
– 50
– 37
– 25
– 20
– 15
– 10
Ladder
Total proteins

Figure S4

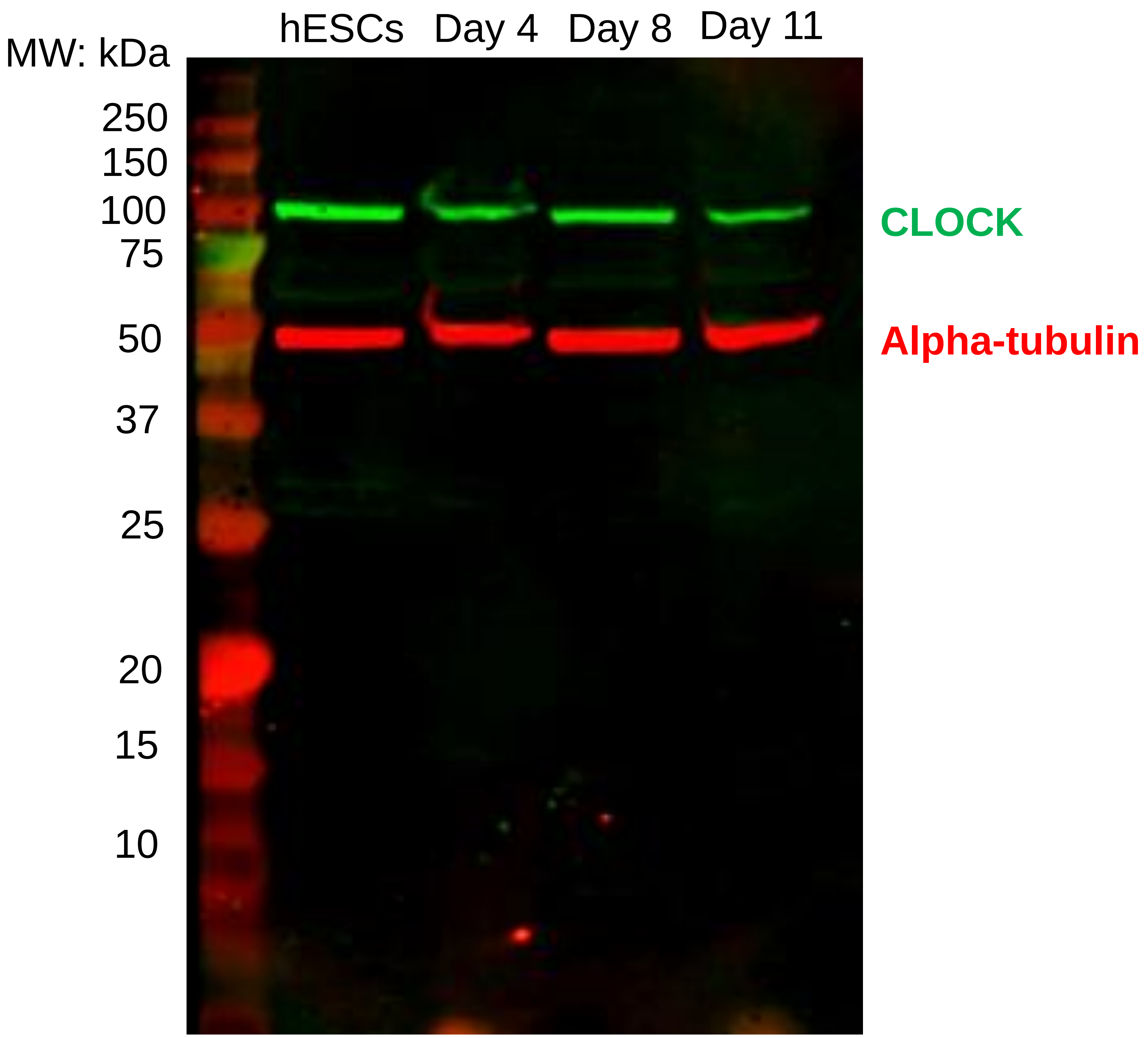

MW: kDa
hESCs
Day 4
Day 8
Day 11
250
150
100
75
50
37
25
20
15
10
CLOCK
Alpha-tubulin

Figure S5

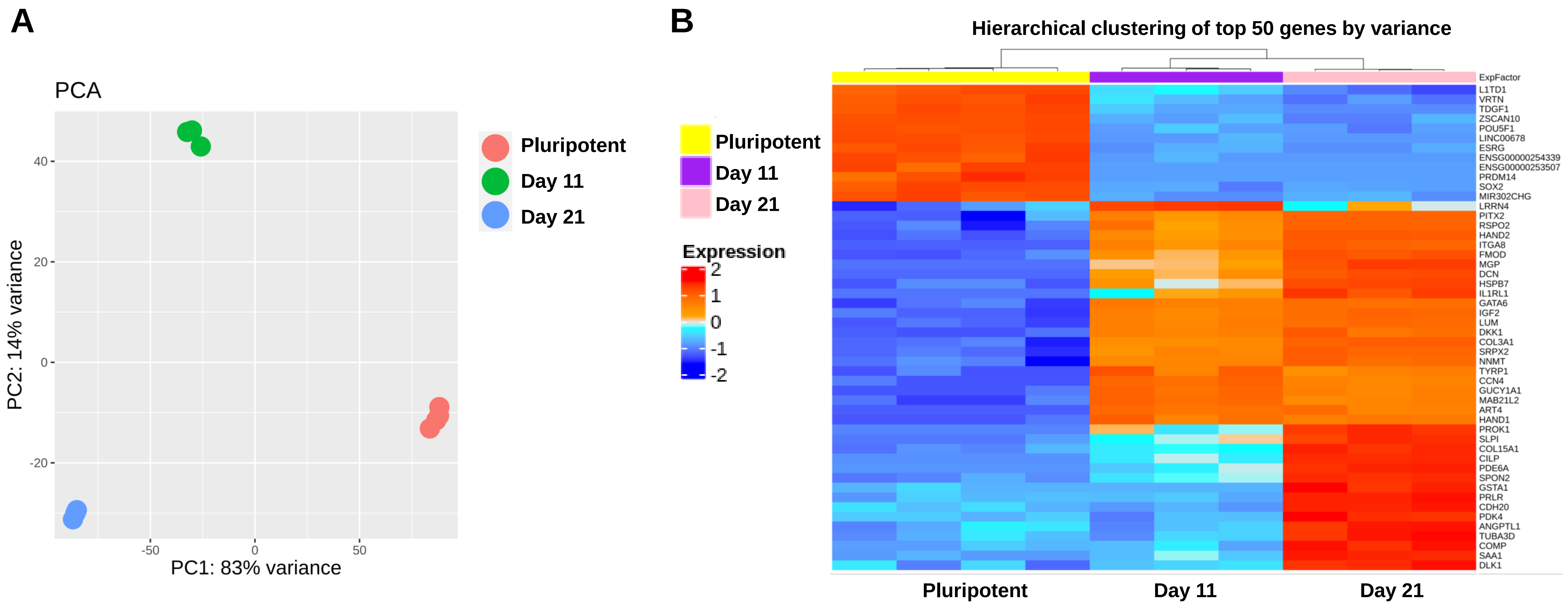
A
PCA
40
20
0
-20
-50
0
50
PC2: 14% variance
PC1: 83% variance
Pluripotent
Day 11
Day 21
B
Hierarchical clustering of top 50 genes by variance
Pluripotent
Day 11
Day 21
Expression
2
1
0
-1
-2
ExpFactor
L1TD1
VRTN
TDGF1
ZSCAN10
POU5F1
LINC00678
ESRG
ENSG00000254339
ENSG00000253507
PRDM14
SOX2
MIR302CHG
LRRN4
PITX2
RSPO2
HAND2
ITGA8
FMOD
MGP
DCN
HSPB7
IL1RL1
GATA6
IGF2
LUM
DKK1
COL3A1
SRPX2
NNMT
TYRP1
CCN4
GUCY1A1
MAB21L2
ART4
HAND1
PROK1
SLPI
COL15A1
CILP
PDE6A
SPON2
GSTA1
PRLR
CDH20
PDK4
ANGPTL1
TUBA3D
COMP
SAA1
DLK1
Pluripotent
Day 11
Day 21

Figure S6
